# Insertion sequence polymorphism and genomic rearrangements uncover hidden *Wolbachia* diversity in *Drosophila suzukii* and *D. subpulchrella*

**DOI:** 10.1038/s41598-017-13808-z

**Published:** 2017-11-01

**Authors:** Rupinder Kaur, Stefanos Siozios, Wolfgang J. Miller, Omar Rota-Stabelli

**Affiliations:** 1 Department of Sustainable Agro-Ecosystems and Bioresources, Fondazione Edmund Mach, San Michele all’Adige, Italy; 2 Centre of Anatomy and Cell Biology, Medical University of Vienna, Vienna, Austria; 3 Institute of Integrative Biology, Faculty of Health and Life Sciences, University of Liverpool, Liverpool, UK

## Abstract

Ability to distinguish between closely related *Wolbachia* strains is crucial for understanding the evolution of *Wolbachia*-host interactions and the diversity of *Wolbachia*-induced phenotypes. A useful model to tackle these issues is the *Drosophila suzukii – Wolbachia* association. *D*. *suzukii*, a destructive insect pest, harbor a non-CI inducing *Wolbachia* ‘*w*Suz’ closely related to the strong CI-inducing *w*Ri strain. Multi locus sequence typing (MLST) suggests presence of genetic homogeneity across *w*Suz strains infecting European and American *D*. *suzukii* populations, although different *Wolbachia* infection frequencies and host fecundity levels have been observed in both populations. Currently, it is not clear if these differences are due to cryptic *w*Suz polymorphism, host background, geographical factors or a combination of all of them. Here, we have identified geographical diversity in *w*Suz in *D*. *suzukii* populations from different continents using a highly diagnostic set of markers based on insertion sequence (IS) site polymorphism and genomic rearrangements (GR). We further identified inter-strain diversity between *Wolbachia* infecting *D*. *suzukii* and its sister species *D*. *subpulchrella* (*w*Spc). Based on our results, we speculate that discernible *w*Suz variants may associate with different observed host phenotypes, a hypothesis that demands future investigation. More generally, our results demonstrate the utility of IS and GRs in discriminating closely related *Wolbachia* strains.

## Introduction

*Wolbachia* are obligate-intracellular bacteria infecting more than half of the arthropod species^1^. Although they are typically maternally inherited by cladogenic transmission or introgression events, horizontal transmission can also occur between closely or distantly related species^2^. *Wolbachia* can spread and maintain themselves in the host by manipulating host reproductive biology^3^. The most studied manipulating strategy is cytoplasmic incompatibility (CI) that favors infected females to enhance rapid bacterial spread throughout the population^4^. In the absence of or in combination with CI, *Wolbachia* may beneficially affect their hosts’ fitness, for example by providing essential nutrients^5^, increasing stem cell proliferation^6^ and protecting against pathogenic RNA viruses^7–10^. Various studies indicate the presence of multiple *Wolbachia* strains in the same host or of different strains in several populations of the same host, inducing various phenotypes^11–14^. Such a large variety of phenotypes caused by *Wolbachia* within the same or different hosts indicate a complex mechanism behind distinct host-*Wolbachia* interactions. The correct typing of *Wolbachia* strain diversity is, therefore, a prerequisite to correctly understand their biology in a given host.

Various molecular tools based on multi-locus sequence typing (MLST) genes together with the hyper-variable *Wolbachia* surface protein (*wsp*) gene^15–18^ have been successfully used for *Wolbachia* strain typing. *Wolbachia* has been classified in distinct types or strains that can be grouped into at least 16 supergroups (named A–F and H–Q)^19^. It is, however, challenging to distinguish among very closely related bacterial strains using single gene phylogenetic or the MLST system alone due to their limited resolution^15,20–23^. For example, the MLST system was insufficient to discriminate closely related *Wolbachia* strains infecting natural populations of *D*. *melanogaster* ^13,15,18,24^. Moreover, MLST failed to differentiate between *w*Ri, *w*Suz and *w*Spc *Wolbachia* strains harbored by their natural hosts *D*. *simulans*, *D*. *suzukii* and *D*. *subpulchrella* (sister species of *D*. *suzukii)*, respectively^25–27^. However, comparison of *w*Ri (complete genome) and *w*Suz (draft genome) revealed several differences such as Insertion sequence (IS) presence/absence polymorphism and genomic rearrangements (GRs)^25^. Whole genome sequencing (WGS), indeed, maximizes the chances of finding informative characters that are less likely to occur in the few genes sampled by MLST and provides enough information to effectively discriminate between indistinguishable strains^28^. For example, a population genomics study allowed the identification of previously uncharacterized *w*Mel diversity within several *D*. *melanogaster* wild populations^29^. However, WGS can be time consuming and expensive for large-scale population genetic studies.

Using a different approach, Riegler and colleagues^13,30^ applied a set of hyper-variable markers based on site polymorphism of IS elements, variable number tandem repeat (VNTR) loci, and chromosomal inversions to discriminate closely related A-supergroup *Wolbachia* strains. IS elements are bacterial class-II transposons of discrete DNA segments that can replicate and spread in the genome through a cut-and-paste mechanism as reviewed in^31^. The majority of IS elements are bound by short terminal inverted repeat (TIRs) sequences of variable lengths that are repeated in opposite orientations at the 5’ and 3’ ends of these elements. ISs are classified into about 20 families on the basis of several conserved features within families, such as structure, insertion site preference, sequence organization, and similar TIRs^31,32^. Together with TIRs, these elements can also undergo ectopic (non-allelic homologous) recombination events resulting in GRs. The genomes of *Wolbachia*, in particular, display a very high number of IS elements representing about 10% of the bacterial genome^33^. These elements can exhibit a large amount of variability in their genomic content and have thus been proven very useful for discriminating very closely related bacterial strains^13,33–37^.

According to MLST, different populations of *D*. *suzukii* harbor the same *w*Suz strain, which in turn is indistinguishable from the new strain (*w*Spc) harbored by *D*. *subpulchrella* ^26,27^. Contrary to their closely related *w*Ri strain that causes strong CI in *D*. *simulans*, *w*Suz and *w*Spc have been characterized by either very low or a complete lack of CI-inducing capability^26,27^. We have previously detected differences in *w*Suz prevalence (and to a lesser extent its CI inducibility) in different *D*. *suzukii* populations. European (EU) *w*Suz infection frequencies are three times significantly higher compared to American (US) ones^27^. Both populations have been reported inducing no considerable CI^26,27^, but EU (French) *D*. *suzukii* reportedly showed a lower, although statistically insignificant, hatch rate in the CI cross^27^. If *D*. *suzukii* actually harbors a single strain of ‘*w*Suz’, we should assume that observed differences in their natural infection prevalence and CI levels are either dependent on the host genetic background or caused by other environmental factors such as temperature or exposure to insecticides^27^. Alternatively, there may exist slightly different cryptic variants of *w*Suz in nature affecting variable levels of their persistence ability in various *D*. *suzukii* populations, but have not yet been distinguished based on standard MLST typing method. Unsuccessful determination of hidden *w*Suz diversity may, therefore, under-estimate the actual biological complexities behind *w*Suz-*D*. *suzukii* interactions.

Our previous comparison of *w*Ri and *w*Suz genomes have provided a putative diagnostic set of markers based on IS site polymorphism and genomic rearrangements^25^. In this study, we validated these diagnostic markers using PCR and Sanger sequencing and revealed an a) intra-strain diversity within *w*Suz from different *D*. *suzukii* populations worldwide and b) inter-strain *Wolbachia* diversity between previously (MLST-based) indistinguishable *w*Suz and *w*Spc strains. These findings will aid in our understanding of *Wolbachia* diversity and infection dynamics within and between *D*. *suzukii* populations and related species. We also discuss the potential implications of *w*Suz geographical diversity in symbiont-based pest management programs.

## Results

**Figure 1:**
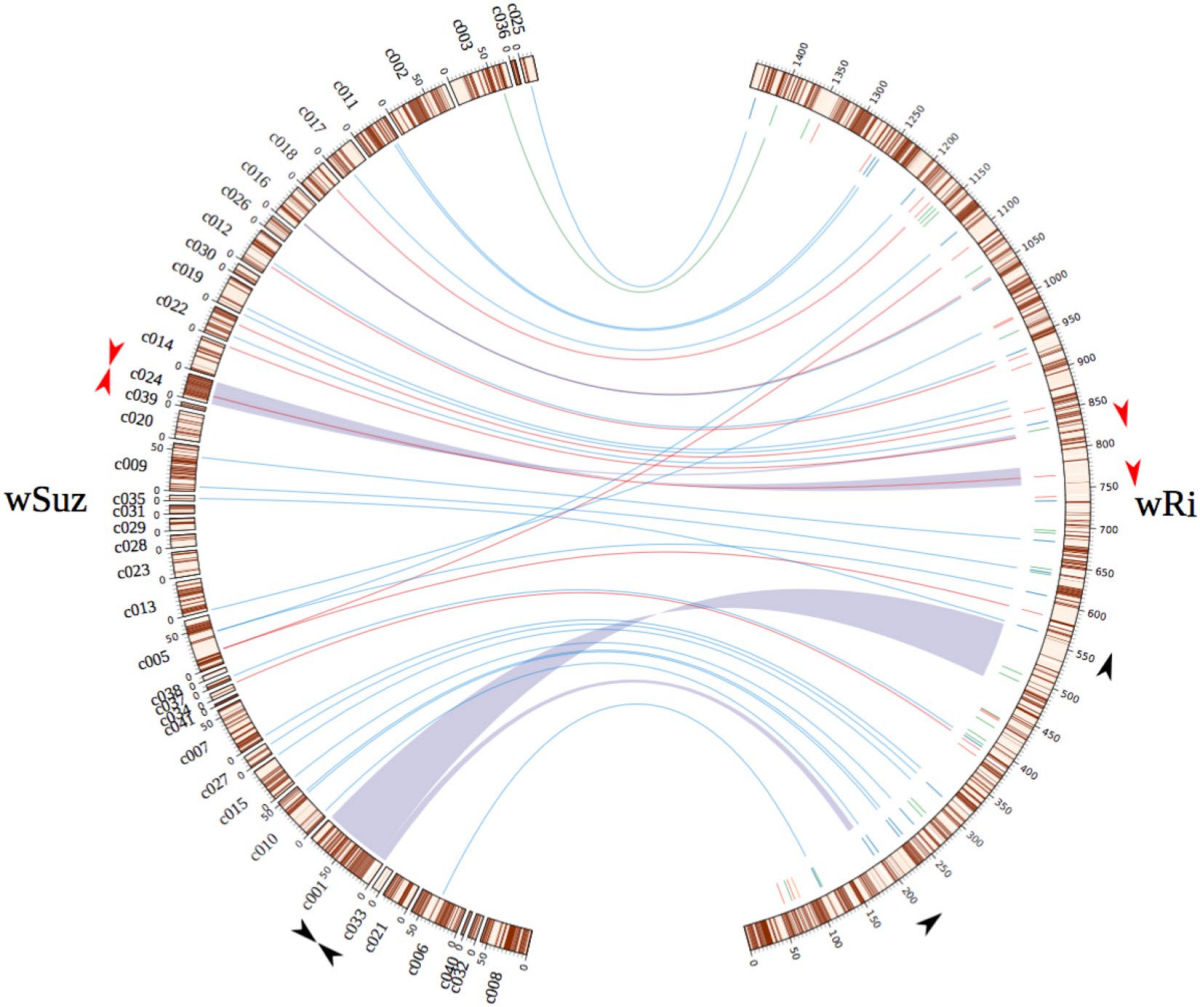
Genome comparison of *w*Suz and *w*Ri for candidate marker loci selection. *w*Suz contigs have been oriented according to *w*Ri genome. Annotated CDSs in either plus or minus strand are represented with brown and cream colored boxes respectively. The grey twisted ribbons represent the two genomic rearrangements detected in *w*Suz relative to *w*Ri. The orientation of the primers used for validating GR1 and GR2 in both genomes are represented with black and red arrowheads respectively. The inner circle in *w*Ri genome represents annotated IS elements, color-coded based on their group affiliation (red: ISWpi1, green:ISWpi2, orange:ISWpi4, blue: ISWpi5 and purple: ISWpi7). Colored lines linking *w*Ri to *w*Suz genome represent the 32 polymorphic IS loci used in the present study. The graph was designed with Circos software^81^.

We selected 32 polymorphic insertion sequence (IS) loci and two large-scale genomic rearrangements (GRs) based on the comparison of *w*Ri and *w*Suz genomes^25^ (Fig. 1). Of the 32 IS-associated loci, eight belonged to ISWpi1 group from the IS5 family, 23 to ISWpi5 group from the IS66 family, and one belonged to ISWpi7 of the IS110 transposon family (listed in Supplementary Table S1). We designed 34 sets of primers and verified these diagnostic markers by PCR amplification (and Sanger-sequencing, when necessary) using genomic DNA extracted from *D*. *simulans*, *D*. *subpulchrella* and two individuals each from thirteen *D*. *suzukii* populations (Table 1). The cumulative results of IS presence-absence polymorphism and the GR based diagnostic PCRs from different *Wolbachia* strains are shown in Table 2.

**Table 1:** Origin of *Drosophila* hosts used in study.

**Host species**	***Wolbachia*** **strain**	**Country of origin**	**Continent**	**Sample status**	**Source location**
*D*. *simulans*	*w*Ri	United States	North America	Live flies	Riverside, CA^73^
*D*. *subpulchrella*	*w*Spc	China	Asia	Live flies	Drosophila Species Stock Center (San Diego, CA, USA)
*D*. *suzukii*	*w*Suz_CHN1	China	Asia	Alcohol-stored	Wenzhou of Zhejiang
*D*. *suzukii*	*w*Suz_CHN2	China	Asia	Alcohol-stored	Weihai, Shandong
*D*. *suzukii*	*w*Suz_JPN1	Japan	Asia	Alcohol-stored	Ehime-fly Stock Center (Kyoto, Japan)
*D*. *suzukii*	*w*Suz_JPN2	Japan	Asia	Alcohol-stored	Ehime-fly Stock Center (Kyoto, Japan)
*D*. *suzukii*	*w*Suz_AUT	Austria	Europe	Live flies	Neustift, Vienna^27^
*D*. *suzukii*	*w*Suz_ITA1	Italy	Europe	Live flies	San Michele all'Adige^27^
*D*. *suzukii*	*w*Suz_ITA2	Italy	Europe	Live flies	Bari^27^
*D*. *suzukii*	*w*Suz_FRA	France	Europe	Live flies	Lyon^27^
*D*. *suzukii*	*w*Suz_GBR	England	Europe	Live flies	Kent^27^
*D*. *suzukii*	*w*Suz_ESP	Spain	Europe	Live flies	Girona^27^
*D*. *suzukii*	*w*Suz_SVN	Slovenia	Europe	Live flies	Izola^27^
*D*. *suzukii*	*w*Suz_USA	United States	North America	Live flies	Oregon
*D*. *suzukii*	*w*Suz_CAN	Canada	North America	Alcohol-stored	British Columbia

**Table 2:** Diagonostic PCR screening of Insertion sequence (IS) site polymorphism and genomic rearrangements (GRs) based markers.

Locus name >>	Insertion sequence (IS) site polymorphism																																Genomic rearrangements (GRs)	
	*w*Ri-specific																														*w*Suz-specific			
	**IS 1**	IS 2	IS 3	IS 4	IS 5	IS 6	IS 7	IS 8	IS 9	IS 10	IS 11	IS 12	IS 13	**IS 14**	IS 15	IS 16	IS 17	IS 18	IS 19	IS 20	IS 21	**IS 22**	IS 23	IS 24	IS 25	IS 26	IS 27	IS 28	IS 29	IS 30	IS 31	IS 32	GR 1	GR 2
*w*Ri	+	+	+	+	+	+	+	+	+	+	+	+	+	+	+	+	+	+	+	+	+	+	+	+	+	+	+	+	+	+	−	−	NA	NA
*w*Spc	+	−	−	−	−	−	−	−	−	−	−	−	−	+	−	−	−	−	−	−	−	+	−	−	−	−	−	−	−	−	−	−	NA	CR
*w*Suz_CHN1	−	−	−	−	−	−	−	−	−	−	−	−	−	−	−	−	−	−	−	−	−	−	−	−	−	−	−	−	−	−	+	−	CR	CR
*w*Suz_CHN2	−	−	−	−	−	−	−	−	−	−	−	−	−	−	−	−	−	−	−	−	−	−	−	−	−	−	−	−	−	−	+	−	CR	CR
*w*Suz_JPN1	−	−	−	−	−	−	−	−	−	−	−	−	−	−	−	−	−	−	−	−	−	−	−	−	−	−	−	−	−	−	+	−	CR	CR
*w*Suz_JPN2	−	−	−	−	−	−	−	−	−	−	−	−	−	−	−	−	−	−	−	−	−	−	−	−	−	−	−	−	−	−	+	−	CR	CR
*w*Suz_AUT	−	−	−	−	−	−	−	−	−	−	−	−	−	−	−	−	−	−	−	−	−	−	−	−	−	−	−	−	−	−	+	+	CR	CR
*w*Suz_ITA1	−	−	−	−	−	−	−	−	−	−	−	−	−	−	−	−	−	−	−	−	−	−	−	−	−	−	−	−	−	−	+	+	CR	CR
*w*Suz_ITA2	−	−	−	−	−	−	−	−	−	−	−	−	−	−	−	−	−	−	−	−	−	−	−	−	−	−	−	−	−	−	+	+	CR	CR
*w*Suz_FRA	−	−	−	−	−	−	−	−	−	−	−	−	−	−	−	−	−	−	−	−	−	−	−	−	−	−	−	−	−	−	+	+	CR	CR
*w*Suz_GBR	−	−	−	−	−	−	−	−	−	−	−	−	−	−	−	−	−	−	−	−	−	−	−	−	−	−	−	−	−	−	+	+	CR	CR
*w*Suz_ESP	−	−	−	−	−	−	−	−	−	−	−	−	−	−	−	−	−	−	−	−	−	−	−	−	−	−	−	−	−	−	+	+	CR	CR
*w*Suz_SVN	−	−	−	−	−	−	−	−	−	−	−	−	−	−	−	−	−	−	−	−	−	−	−	−	−	−	−	−	−	−	+	+	CR	CR
*w*Suz_USA	−	−	−	−	−	−	−	−	−	−	−	−	−	−	−	−−	−	−	−	−	−	−	−	−	−	−	−	−	−	−	+	−	CR	CR°
*w*Suz_CAN	−	−	−	−	−	−	−	−	−	−	−	−	−	−	−	−	−	−	−	−	−	−	−	−	−	−	−	−	−	−	+	−	CR	CR°

῾+’ sign indicates presence and῾−’ indicates absence of the IS element.

Bold letters indicate IS loci shared by *w*Ri and *w*Spc, NA- No amplification, CR- Chromosomal rearrangement.

CR°- Chromosomal rearrangement with size polymorphism.

### IS insertion site polymorphism and genomic rearrangements differentiate *w*Suz, *w*Spc and *w*Ri *Wolbachia* strains

**Figure 2:**
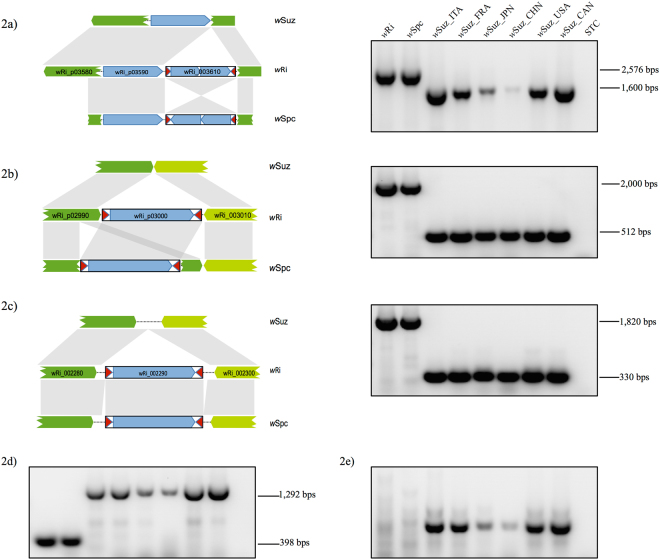
Inter-strain polymorphism between closely related wSuz, wSpc and wRi *Wolbachia* strains. Arrows with different shades of green represent different ORFs while the blue arrows represent transposase genes. Red arrowheads correspond to terminal inverted repeats (TIRs). A–C. *w*Ri-specific IS loci (**a**) IS element present at locus IS1 belonging to ISWpi1 group shows inversion between *w*Ri and *w*Spc, and absence from *w*Suz strain, (**b**) and (**c**) Two IS elements (loci ID- IS14 and IS22) show independent insertion events between *w*Ri and *w*Spc however completely absent from *w*Suz genome. (**d**) *w*Suz-specific IS element (locus ID- IS31) belonging to ISWpi1 group shows insertion only in *w*Suz from all populations producing 1292 bps long amplicon, but absent from *w*Ri and *w*Spc with 398 bps amplicon size. (**e**) Genomic rearrangement (GR1) showing amplification in *w*Suz only, absent in *w*Ri and *w*Spc. Lanes from left: *w*Ri, *w*Spc, *w*Suz_ITA, *w*Suz_FRA, *w*Suz_JPN, *w*Suz_CHN, *w*Suz_USA, *w*Suz_CAN and STC-*Wolbachia* negative control. The full-length gel pictures are presented in Supplementary Figure S2.

Out of the 32 polymorphic IS loci, 27 were specific of *w*Ri (IS2-IS13, IS15-IS21, IS23-IS30), two were specific of *w*Suz (IS31 and IS32), and three were shared between *w*Spc and *w*Ri (IS1, IS14 and IS22). The latter were demonstrated by the similar amplicon sizes in *w*Spc and *w*Ri (2,576bps, 2,000bps and 1,820bps respectively) compared to *w*Suz (1,600bps, 512bps and 330bps respectively) (Fig. 2a–c). Sequence analysis, however, revealed the presence of IS target-site variations at all these three loci (Fig. 2a–c). At IS1 locus, an ISWpi1 (*w*Ri_003610) element was shared, but reversely orientated in *w*Spc and *w*Ri (Fig. 2a). At IS14 and IS22 loci, two ISWpi5 elements (*w*Ri_p03000 and *w*Ri_002290, respectively) were shared among *w*Spc and *w*Ri, but the exact insertion sites differed between the two strains: at IS14, the ISWpi5 element in *w*Spc was inserted 84bp upstream relative to *w*Ri, whilst for the IS22 locus, the insertion in *w*Spc was 8bp downstream to that of *w*Ri (Fig. 2b & c).

**Figure 3:**
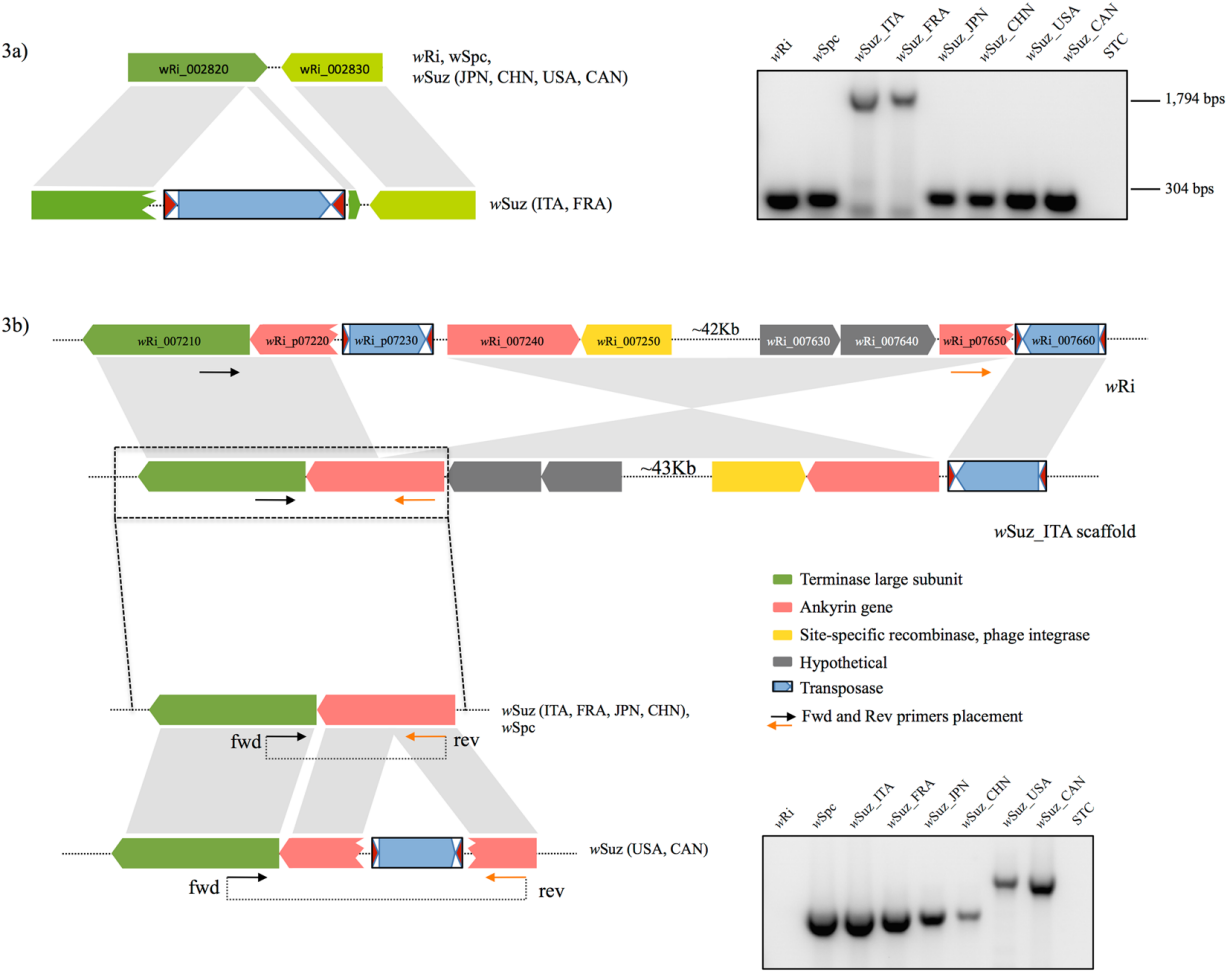
Intra-strain polymorphism of *w*Suz within different *D. suzukii* populations. (**a**) *w*Suz specific IS element (Locus ID- IS32) showing 1,794 bps amplicon size polymorphism in European *w*Suz (*w*Suz_ITA and *w*Suz_FRA) strains in comparison to other *w*Suz strains showing amplification of 304 bp size, similar to *w*Ri and *w*Spc. (**b**) Genomic rearrangement (GR2) showing size polymorphism in American (USA) and Canadian (CAN) *D*. *suzukii* only. Upper panel - schematic diagram of Inverted translocation (IT) shown in *w*Ri and *w*Suz genome. The full-length gel pictures are presented in Supplementary Figure S2.

Two large-scale genome rearrangements (GR1 and GR2) further discriminated *w*Suz, *w*Spc and *w*Ri (Table 2). Primers flanking both GR regions in *w*Suz (Fig. 1, Supplementary Table S1) were used to confirm the rearrangements using PCR: GR1 was confirmed as a genomic inversion in all *w*Suz variants compared to *w*Spc and *w*Ri (Fig. 2e); GR2 was inverted in both *w*Suz and *w*Spc, but not in *w*Ri (Fig. 3b).

### Polymorphism in *w*Suz strains from different *D*. *suzukii* host populations

We detected intra-strain polymorphism within *w*Suz strains from different *D*. *suzukii* populations listed in Table 1. Hereafter, several *D*. *suzukii* populations from different countries, but of the same continent, have been referred by their continental names. A *w*Suz-specific IS element at locus IS32 was exclusively found in European samples (*w*Suz_ITA, *w*Suz_FRA), and not in American (*w*Suz_USA, *w*Suz_CAN) and Asian (*w*Suz_CHN, *w*Suz_JPN) populations (Table 2, Fig. 3a). Sequence analysis further confirmed that IS32 belongs to the ISWpi5 group and is inserted six nucleotides upstream to the stop codon of a gene homologous to *w*Ri_002820. The *w*Ri_002820 homologues in *w*Ri, *w*Spc and non-European *w*Suz strain variants remained intact and coded for a hypothetical protein^33^, however, we detected low similarities to the SMC (Structural Maintenance of Chromosomes) protein family. The ISWpi5 insertion in European *w*Suz variant at the same locus resulted in 9 extra amino acids addition at the C-terminus of the protein due to in-frame position of left TIR of the IS element (Supplementary Fig. S1).

Sequence comparison of GR2 showed that this inverted region, spanning more than 40Kb in size, is flanked by two nearly identical ISWpi7 elements (*w*Ri_p07230, *w*Ri_007660) and results in the truncation of an ankyrin (ANK) gene represented by two pseudogenes *w*Ri_p07220 and *w*Ri_p07650 flanking the inversion in *w*Ri genome (Fig. 3b). In contrast, the ANK gene was intact in *w*Spc and all of the *w*Suz variants, except for those infecting American *D*. *suzukii* (*w*Suz_USA and *w*Suz_CAN), where a similar ISWpi7 element truncated the ANK gene causing no inversion as confirmed by PCR and Sanger sequencing (Fig. 3b). Overall, IS- and GR- based diagnostic markers revealed the existence of at least three different *w*Suz genotypes infecting *D*. *suzukii* populations from American, Asian and European continents.

### Phylogenetic analyses recapitulate genomic differences

**Figure 4:**
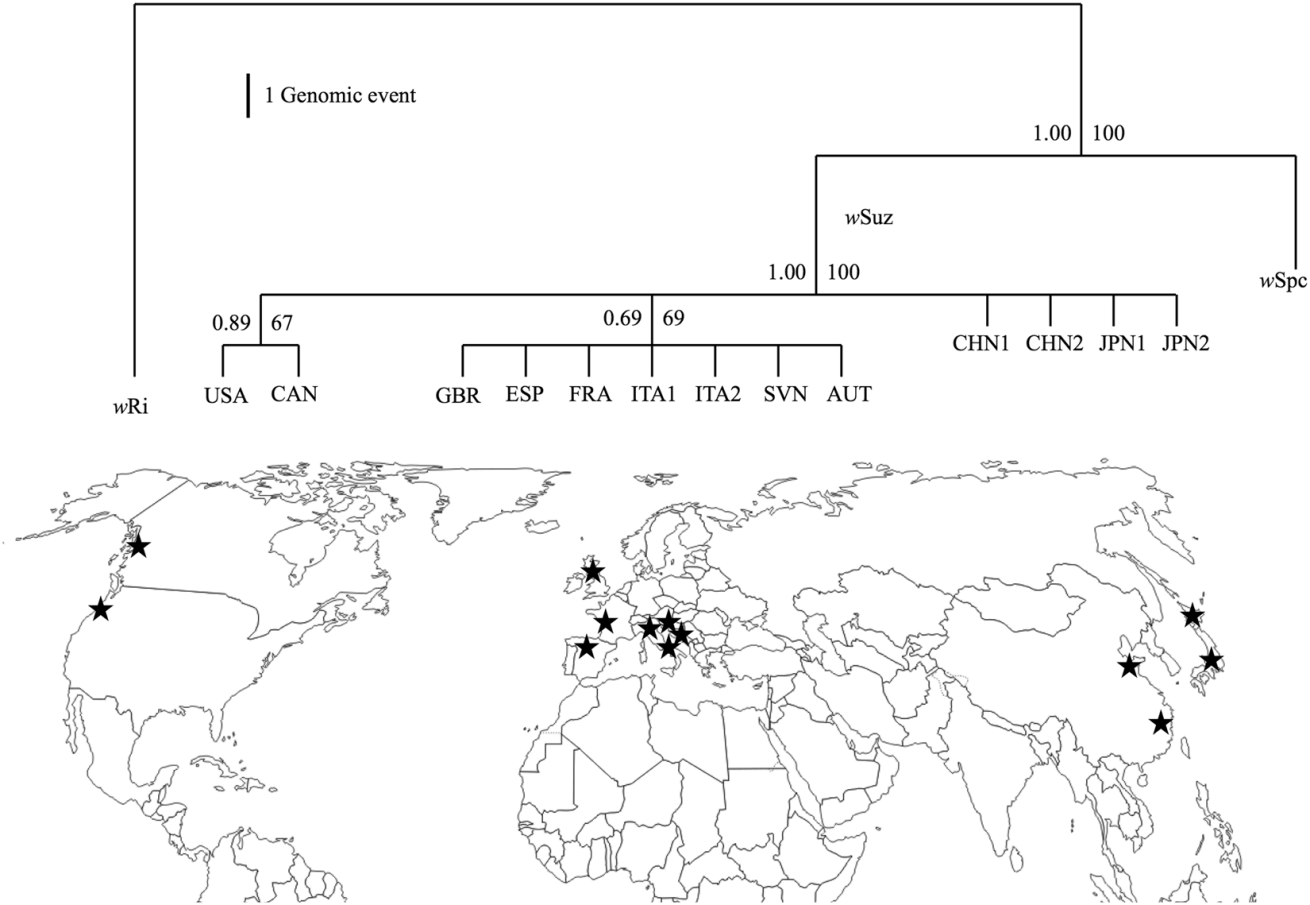
Phylogeny from all polymorphic loci. Cladogram of *w*Ri, *w*Spc and *w*Suz *Wolbachia* strains inferred from the 34 character- state matrix. Support values for each node are placed, on the left is the Bayesian posterior probability and right is the percentage bootstrap support from TNT based parsimony analysis. Black stars on the map represent each sampled population of *D*. *suzukii* used in this study. The map was modified from d-maps.com (http://d-maps.com/m/world/centreeurope/centreeurope22.gif).

On the basis of our IS and GR strain typing patterns, we constructed a character-state matrix (Supplementary Table S2) and performed phylogenetic analysis. Maximum parsimony and Bayesian analysis resulted in identical tree topologies (Fig. 4). *w*Suz strains were found clearly monophyletic: European and American *w*Suz genotypes originated independently from a more ancestral Asian infection although with weak support values due to relatively few synapomorphic characters available to compute phylogeny.

## Discussion

Identification and discrimination of “cryptic” (not yet discovered and very closely related) *Wolbachia* genotypes is essential to understand the biology and the evolution of host-*Wolbachia* associations. Previous screenings based on MLST failed to discriminate between *w*Suz (harbored by *D*. *suzukii*), *w*Spc (*D*. *subpulchrella*) and *w*Ri (*D*. *simulans*) *Wolbachia* strains, suggesting the presence of a monomorphic *Wolbachia* infecting different host species^26,27^. The same studies suggested the absence of genetic polymorphism in *Wolbachia* infecting different *D*. *suzukii* populations. Indeed, whole genome comparison of *w*Ri and *w*Suz strains revealed extensive sequence similarity between the two *Wolbachia* strains^25,38,39^ indicating that *w*Ri and *w*Suz are very closely related and diverged very recently. Moreover, the newly released draft genome of *w*Spc strain indicated a closer relationship between *w*Suz and *w*Spc^40^ (pre-print, https://doi.org/10.1101/135475). Despite the high level of similarity, *w*Ri and *w*Suz differed substantially in terms of their insertion sequence (IS) site polymorphism and genomic rearrangements (GRs). In this study, we have shown the utility of these polymorphic markers to distinguish *w*Spc from *w*Ri and *w*Suz, as well as to identify intra-strain *w*Suz diversity among different continental populations of *D*. *suzukii* (from America, Asia and Europe).

We first detected target site variations as well as sequence inversion of IS elements at the three loci (IS1, IS14 and IS22) shared between *w*Ri and *w*Spc. IS element inversions have previously been reported in *Wolbachia* and attributed to the effect of ectopic recombination between the TIRs of IS elements^41^. In case of the IS1 locus, ectopic recombination has presumably resulted in the complete inversion of the insertion element including the asymmetric TIRs in *w*Ri and *w*Spc. Furthermore, target site polymorphism was detected in case of IS14 and IS22 loci in *w*Spc compared to *w*Ri. Both cases involved the insertion of an ISWpi5 element, a member of the IS66 family. Shared insertions of the same IS element at slightly different sites suggests possible independent insertion events in the two strains; however, it is not clear whether IS elements of the IS66 family exhibit sequence-specific or region-specific target preference^42,43^. An alternate parsimonious scenario would be that the observed target site polymorphism is the result of IS excision and local re-integration in either *w*Ri or *w*Spc genomes after their divergence from a common ancestral genotype. Our results have practical implications for improving IS polymorphism-based *Wolbachia* strain typing methodologies. Many of the previous studies focus on simple PCR amplicon size polymorphism detection (presence/absence patterns) by gel electrophoresis^13,30,35^. We, however, advocate that for obtaining higher resolution strain typing, sequencing of the IS element as well as the respective insertion site is also important to uncover orientation- or target site-based variations, which otherwise can be neglected due to the similar PCR amplicon size obtained.

We further detected intra-strain *Wolbachia* polymorphism in *w*Suz strain from different geographical populations of *D*. *suzukii* host. Historically originating from Asia, *D*. *suzukii* has recently invaded Europe and America^44,45^. Population studies suggested that the two continents were invaded independently from two distinct Asian regions^46,47^. The presence of geographical diversity in *w*Suz *Wolbachia* strains (Fig. 4) is in agreement with this scenario, suggesting that founding *D*. *suzukii* individuals carried different *w*Suz variants in each of the two continents. We cannot exclude, however, the effects of environmental constraints that may have triggered rapid genomic changes in *Wolbachia* either due to adaptation and/or relaxed selection in a new environment. For example, a rapid adaptive evolution of *w*Mel-Pop strain of *D*. *melanogaster* has been previously reported after its artificial transfer to *Aedes aegypti* mosquito cell lines^48^. Another study showed altered behavior of *Wolbachia* when passaged for several generations through heterozygous mutant lines of *D*. *melanogaster* ^49^. Concurrent with this, it has been suggested that the IS mobility is able to promote the evolutionary adaptation of their hosts^50,51^. However, a different study pointed out that cryptic and low-titer *Wolbachia* infections within or between host populations can shift in prevalence during strong bottleneck events, for example during artificial host transfers^52^. Under these scenarios, an alternative explanation for the geographical diversity of *w*Suz could be that the different *w*Suz genotypes may initially coexisted in the native Asian populations of *D*. *suzukii* as low-titer or rare variants within or between populations and during its colonization of America and Europe, *D*. *suzukii* might have experienced a mixture of bottlenecks^46,47^ and differential selective pressures in the two continents to evolve into new genotypes. More *D*. *suzukii* samples from other Asian populations and at more time points will be needed in order to test this hypothesis.

The presence of *w*Suz variants among different *D*. *suzukii* populations raises another interesting question as to what extent this genetic diversity could be associated with phenotypic variations in the host. Earlier studies on *Wolbachia* from European and American *D*. *suzukii* populations revealed that *w*Suz does not induce significant levels of CI and is imperfectly maternally transmitted from the mother to the progeny^26,27^. To maintain its infection status in the wild, CI is often considered as the driving force for *Wolbachia*-mediated sweeps in insect host populations^53^; the persistence of *w*Suz despite inducing no apparent CI under laboratory conditions in both continental populations points towards some positive fitness effects. Indeed, experimental data show *w*Suz-mediated high fecundity^54^ and strong protection against RNA viruses^55^ in *D*. *suzukii*. However, these fitness advantages are not conserved among different populations of *D*. *suzukii*: first, *w*Suz infection is higher prevalent and provides with more fecundity in European populations than in American ones^26,27,54^; second, we detected higher *w*Suz anti-viral protection ability in the American population than in the European one (Kaur R., Martinez J., Jiggins F., Rota-Stabelli O., Miller W.J., Tissue-specificity of *Wolbachia* in *Drosophila* vary in their interactions towards *Drosophila* C Virus and Flock House Virus, *manuscript in preparation*). Because the symbiont strain rather than the host genetic background has been demonstrated to determine the degree of *Wolbachia*-mediated antiviral protection effect^56^, we speculate that the observed differences in the antiviral protection (and perhaps fecundity and infection frequency) may be, at least partially, attributed to the different *w*Suz genotypes we have detected. It is important to stress that *Wolbachia*-induced phenotypes depend not only on the *Wolbachia* genetic background but also on the genetic background of the host^57^ and, more importantly, on the host-*Wolbachia* associations^58–60^. Indeed, population studies indicated a certain degree of genetic diversity between European and American *D*. *suzukii* ^46,47,61^. It is therefore highly plausible that a rather complex series of interactions took place in the European and American *D*. *suzukii-w*Suz systems, leading to observed differences.

One of the most interesting genomic events we have found is at the IS32 locus where the insertion element is exclusively present in European *w*Suz variant (Fig. 3a), making it a highly diagnostic marker for characterizing *w*Suz intra-strain diversity. This insertion terminally disrupts the ORF of a *Wolbachia* gene named *w*Ri_002820, likely encoding a protein involved in tRNA synthesis, DNA repair and chromosomal segregation in *w*Au *Wolbachia* strain^62^. Another interesting event is the large-scale genomic rearrangement - GR2, flanked by two nearly identical inverted repeat elements in *w*Ri genome. Similar genomic events associated with flanking inverted or direct repeats have previously been detected in other *Wolbachia* strains, e.g. *w*MelPop, giving rise to large-scale inversions^48,63^ or extensively amplifying Octomom locus^64^ respectively, and differentiating it from closely related *w*Mel strain. GR2 is, therefore, another diagnostic marker for screening *w*Suz genotypes since the 5’-flanking inverted IS element resulting in GR2 is found in American *w*Suz only. This IS element, similar to *w*Ri, results in truncation of an Ankyrin (ANK) repeat domain protein, but without causing an inversion (Fig. 3b), suggesting that this chromosomal inversion event is specific to *w*Ri only. Furthermore, it is known that such insertion/truncation events may cause gene inactivation or alter gene regulation and expression^50,65^ resulting in potential phenotypic changes. Proteins with eukaryotic domains such as ANK repeats are considered primary candidates for mediating host-*Wolbachia* interactions; variability in ANK repeat structure and number could affect the affinity, specificity, localization, expression and function of these ANK proteins^66,67^. Thus, we prudently hypothesize that the structural variability of these proteins in *w*Suz variants might be associated with different observed inter-continental phenotypes and host-*Wolbachia* associations in *D*. *suzukii*. Life trait experiments involving American-European *D*. *suzukii* cross infections should be performed to verify our working hypothesis.

We finally discuss the potential implications of genetic diversity found in *D*. *suzukii* (and *D*. *subpulchrella*) for *Wolbachia*-based pest management programs. *Wolbachia* is a promising tool for developing control strategies of arthropod pest populations based on the CI phenotype^68,69^. Previous studies have shown no CI inducing capability in Italian, French, East and West US coast *D*. *suzukii* populations^26,27^. In addition *w*Spc, similar to *w*Suz, does not induce CI in its native host *D*. *subpulchrella* ^26^. However, the aforementioned closely related *w*Suz, *w*Spc and *w*Ri strains could have quite different effects on the host biology, if transfected or introgressed in a different host system. Various experiments have been carried out successfully to test this cross-compatibility hypothesis, with artificial transinfection of CI-inducing *Wolbachia* among several *Drosophila* species both intra-^14^ and inter-specifically^70,71^. Future experiments involving artificial transinfection or introgression of *D*. *suzukii* with closely related *Wolbachia* strains such as *w*Spc or *w*Ri can be performed in order to assess their modification and rescue capabilities to aid the development of bi-directional CI-based pest control programs^72,73^. Moreover, a correlation between IS-distinctive *w*Pip *Wolbachia* genetic variants and CI crossing types has been shown in *Culex pipiens* mosquito populations^35,74,75^. We propose that different geographical *D*. *suzukii* populations harboring *w*Suz variants should be inter-crossed to better explore the host-*Wolbachia* genetic background effects on CI-induction.

## Methods

### Fly strains and rearing

Details of different *Drosophila-Wolbachia* associations assayed in this study as well as their sources and origin are listed in Table 1. All live flies were maintained on standard fly food in vials at a constant temperature of 22°C with a 12:12 light:dark cycle.

### Candidate marker loci selection

We previously detected several structural variations such as insertion sequence (IS) site polymorphism and genomic rearrangements (GRs) separating *w*Suz from the close-related *w*Ri strain^25^. A total of 34 candidate markers including 32 IS site polymorphic loci together with two large-scale GRs were chosen to study previously uncharacterized inter- and intra-strain *Wolbachia* polymorphism (Fig. 1). Primers were designed on their respective 5′ and 3′ flanking regions using Primer 3^76^ as implemented in Geneious software version 7.0.6 (Biomatters, New Zealand). Primer sequences are listed in Table S1. Conserved protein domains of diagnostic IS target genes were identified using the NCBI’s conserved domain database in conjunction with BlastP and also independently verified using EMBL-EBI’s InterProScan^77^ and Pfam^78^. BlastP analysis was conducted using the NCBI BlastP program.

### PCR amplification and sequencing

Host genomic DNAs were extracted using DNeasy tissue kit (Qiagen) according to the manufacturer’s instructions. Diagnostic PCR assays were performed in 20 μl reaction mixtures containing 1x GoTaq reaction buffer, 3.0 mM MgCl_2_, 0.5 μM of forward and reverse primer, 35 μM dNTPs, 1U of *Taq* Polymerase (Promega) and 30–50ng of DNA template. PCR amplification was performed on a BioRad Thermal Cycler using the following thermal profiles: 1 cycle (94°C for 3 min), 35 cycles (94°C for 30 sec, 60°C for 30 sec, 72°C for 1 min) and 1 cycle (72°C for 8 mins). Amplicons were examined using gel-electrophoresis on 1% Agarose gel stained with ethidium bromide. Gel images were visualized using an ultraviolet gel documentation system (iNTAS, Goettingen, Germany). Images were cropped to remove extraneous gel area. The Qiagen® Nucleotide Removal Kit was used to purify the reaction products, followed by Sanger sequencing analysis. All sequences have been deposited in Genbank under accession numbers MF034744 – MF034749.

### Phylogenetic analysis

We conducted Parsimony and Bayesian analyses on a character state matrix in which each genomic locus listed in Table S1 was considered as an independent character. The presence/absence pattern of the characters was deduced directly from the amplified PCR bands of two individuals from each population. Presence of insertion sequence was designated with 1, and absence with 0. Whenever an IS element at a defined insertion locus was of a different size than expected, it was designated with a number higher than 1. Parsimony analysis was performed in TNT (Tree analysis using New Technology) program v1.5^79^ by implementing traditional TBR (tree bisection reconnection) heuristic search algorithm, using 1000 replicates, saving 10 trees per replicate and replacing existing trees. To assess confidence in the resulting phylogenetic estimate, the data were subjected to a bootstrap using symmetric resampling^79^ and a search with 33% change probability (100 replicates), and jackknife analysis using a traditional search with a 36% removal probability replicated 5,000 times. Bayesian phylogenetic analysis was performed with MrBayes v3.2.5^80^ using the Mk model of Lewis (2001) with the assumption that only characters that varied among taxa were included (i.e. coding = variable). Two simultaneous iterations of the Bayesian analysis were run using four simultaneous Monte Carlo Markov Chains (MCMC) for 1,000,000 generations. Trees were sampled every 100 generations. Posterior probabilities representing a measure of clade credibility were generated from the majority-rule tree composed from trees sampled from both runs, after excluding the first 25% of trees as burn-in.

### Data Availability

All data generated or analyzed during this study are included in this article (and related Supplementary information files).

## Electronic supplementary material

Supplementary information
