# Supplementary material for: Insertion sequence polymorphism and genomic rearrangements uncover hidden Wolbachia diversity in Drosophila suzukii and D. subpulchrella
Source: Sci Rep. 2017 Nov 1;7:14815. doi: 10.1038/s41598-017-13808-z (PMC5665950; doi:10.1038/s41598-017-13808-z)
Supplement: Supplementary file 1 — Supplementary information [file 41598_2017_13808_MOESM1_ESM.pdf]

Insertion sequence polymorphism and genomic rearrangements uncover hidden *Wolbachia* diversity in *Drosophila suzukii* and *D. subpulchrella*

Rupinder Kaur[1, 2*], Stefanos Siozios[3*], Wolfgang J. Miller[2], Omar Rota-Stabelli[1@]

Table S1. Primers sequences of Insertion sequence (IS) site polymorphism and Genomic rearrangement (GR) based markers

IS family	Annotation	Locus ID	Primer name	Primer sequences (5' => 3')	Coordinates in *w*Ri genome	Estimated size in *w*Ri (bps)	Estimated size in *w*Suz (bps)
IS5 family	ISWpi1	IS1	ISpol1F<br>ISpol1R	TTCGCTACCCTGTTGTTCCC<br>GGTTCAAGCTAAACACCGCG	385903-388478	2576	1600
	ISWpi1	IS2	ISpol2F<br>ISpol2R	ACTGCGTAGACGTTCCTGTG<br>GTAGGGGCTGCAAGGCTTAA	765749-767702	1954	1035
	ISWpi1	IS3	ISpol3F<br>ISpol3R	TCGTGCAACTCCTGGTCTTC<br>GTGGGACGAGCAACAAGAGA	855201-856998	1798	879
	ISWpi1	IS4	ISpol4F<br>ISpol4R	TTGAATGTGTGCCAAACCGC<br>CGTTGCTCTTGGTTGGTGTG	929665-931529	1865	946
	ISWpi1	IS5	ISpol5F<br>ISpol5R	ACGCCTTAAATGGGGCTTCA<br>TTTAGTCGAAGCACGCCTGT	1041226-1043005	1780	861
	ISWpi1	IS6	ISpol6F<br>ISpol6R	TGAACTGCCGCGTTCTAACT<br>GTGGGTAACACTGGGTTCGA	1166783-1168253	1471	552
	ISWpi1	IS7	ISpol7F<br>ISpol7R	ACCGCATGCGTCTCTTCCCC<br>TGCACACCGCTGCACCTTGC	582773-584203 /<br>1089232-1090662	1431	512
	ISWpi1	IS31<br>(*w*Suz-specific)	ISpol31F<br>ISpol31R	GGGCTTTTTGCGAGTGTAGCTGT<br>CATCTTGCAGCAGGAGTGTCGT	823636-824033	398	1292
IS66 family	ISWpi5	IS8	ISpol8F<br>ISpol8R	ACCAGTATTGATGCTGCGCT<br>TGCAAAGCACAGAAACCTTCG	679259-681204	1946	456

ISWpi5	IS9	ISpol9F<br>ISpol9R	GCAGTTTCTGGTTCTGGTGC<br>TAGTGCAGCATCTTGTGGCA	837678-839632	1955	330
ISWpi5	IS10	ISpol10F<br>ISpol10R	CCGCTAGCCCCCTTATCATG<br>CACACGCCCGCTATTCCTAT	936354-938508	2155	665
ISWpi5	IS11	ISpol11F<br>ISpol11R	GATACTGCGGCGGTATGACA<br>TAATCGCTATGCTCCTGCCG	1192806-1194739	1934	444
ISWpi5	IS12	ISpol12F<br>ISpol12R	GGGCAGATTACCGATCAGGG<br>TTAAGCAAGAGGTCGCAGCA	1253149-1255132	1984	494
ISWpi5	IS13	ISpol13F<br>ISpol13R	ACACTGGGATGACAAACGGT<br>GGTGCTGCAATGCGTAAGTT	1258576-1260575	2000	510
ISWpi5	IS14	ISpol14F<br>ISpol14R	AGCCAAGTTGTCACCTTCTGT<br>GGTCACCAGCCAGGAAAAAG	317383-319382	2000	512
ISWpi5	IS15	ISpol15F<br>ISpol15R	CTGACCCTGAAAGCACTGGT<br>GAGGGCAATAGCAGAACCGA	866390-868283	1894	264
ISWpi5	IS16	ISpol16F<br>ISpol16R	TGAGCACGATCCTGAAAAGGT<br>AACGCACCTACGGTATAGCG	877110-879053	1944	317
ISWpi5	IS17	ISpol17F<br>ISpol17R	CGCTCTGGTTGGCAAGAAAA<br>CCCTCATCAATATCCACAGGAGT	1038164-1040941	2778	234
ISWpi5	IS18	ISpol18F<br>ISpol18R	TGCTGCTCGCATAAATTCTGCCTGT<br>GTGGCAACCGATGCGCACTT	980518-982570	2028	357
ISWpi5	IS19	ISpol19F<br>ISpol19R	TCGCATCAGCTTCACCCTCCCT<br>TCCTGTGTTTTTACGCGTGTTTCCA	198153-200132	1980	490
ISWpi5	IS20	ISpol20F<br>ISpol20R	ACGTATTCGTGATGAACGGGCTGT<br>ACAGCAAGATGCGAAGGAGAGACT	638019-639891	1873	383

	ISWpi5	IS21	ISpol21F<br>ISpol21R	CCCTTCTTGCTGCTCTTCCTTTGGC<br>TTTGCTTGATACTGGAGCAACCGAT	231991-233923	1933	443
	ISWpi5	IS22	ISpol22F<br>ISpol22R	AGCCAGCAAGCACAAGGTGTCA<br>GCATGGATGTTGCGATTCTCAAGGC	237160-238979	1820	330
	ISWpi5	IS23	ISpol23F<br>ISpol23R	TGCTTGGTAGGTAGATGTGTGCAAG<br>AAGTGCAATAACGGTCATACCGCA	256923-258881	1959	469
	ISWpi5	IS24	ISpol24F<br>ISpol24R	CAGCGGCAGCACTCCCAACA<br>AGCCAACCACGGGGCTTCTT	308087-310060	1974	484
	ISWpi5	IS25	ISpol25F<br>ISpol25R	CCAGCGTCACGCGCTGGAAT<br>GCTACCACGAGCGGCACTGA	560111-562072	1962	472
	ISWpi5	IS26	ISpol26F<br>ISpol26R	TGCCATGGTGCGGTTACTGTGG<br>ACAGCAGGAGTTGCTGTGGGT	391590-393346	1757	270
	ISWpi5	IS27	ISpol27F<br>ISpol27R	TTAGCCAACCACGAGGCTTC<br>GGATGTCATTCCAGTGCCCA	110357-112124	1768	278
	ISWpi5	IS28	ISpol28F<br>ISpol28R	CCTATAGCTATACCGCCGCG<br>GTTGCTGCTCTTTTTCGGCA	1430759-1435759	4892	720
	ISWpi5	IS29	ISpol29F<br>ISpol29R	ACGGTGGGGGTTCTCTTGGCA<br>ACCCTTTAGCACCTGTAATGCCCT	607466-609421 /<br>1113925-1115880	1959	466
	ISWpi5	IS32<br>(*w*Suz-specific)	ISpol32F<br>ISpol32R	TGGGTCTTGCTGCTGGACTGA<br>ACATATCGCGCAGTCGGCAC	296839-297132	304	1794
IS110 family	ISWpi7	IS30	ISpol30F<br>ISpol30R	TGATGGAGTGGAGCATGCAG<br>TCGATGAAGCGGTCCAAACA	1404496-1406245	1750	260

Genomic Rearrangements*

Rearrangement 1	GR1	c65_F	GGGAGAGTAGGTCGCCGCCA	191445 – 191464	no amplification in *w*Ri
		c65_R	AGCCAAGCGACGCAACAAACA	556008 – 556028	
Rearrangement 2	GR2	c151_F	CTGCATTGCGCTATAGAGTTTGA	828612 – 828634	no amplification in *w*Ri
		c151_R	TGGCATAGATCGGAGGCTTAC	779789 – 779809	

* since *w*Suz genome is not closed, we could only validate with PCR one of the breakpoints for each rearrangement.

Table S2. 34 character-state matrix designed on IS- and GR-based *Wolbachia* strain-typing pattern

*w*Ri	1111111011111111111111110111011100
*w*Spc	1000000000000010000000100000000001
*w*Suz_CHN1	0000000100000000000000000000000011
*w*Suz_CHN2	0000000100000000000000000000000011
*w*Suz_JPN1	0000000100000000000000000000000011
*w*Suz_JPN2	0000000100000000000000000000000011
*w*Suz_AUT	0000000100000000000000002000000011
*w*Suz_ITA1	0000000100000000000000002000000011
*w*Suz_ITA2	0000000100000000000000002000000011
*w*Suz_FRA	0000000100000000000000002000000011
*w*Suz_GBR	0000000100000000000000002000000011
*w*Suz_ESP	0000000100000000000000002000000011
*w*Suz_SVN	0000000100000000000000002000000011
*w*Suz_USA	0000000100000000000000000000000013
*w*Suz_CAN	0000000100000000000000000000000013

Figure S1. Transposable element insertion at IS32 locus in *w*SuzEU compared to homologous gene *w*Ri_002820 in *w*Ri genome.
L-TIR: Left Terminal inverted repeat

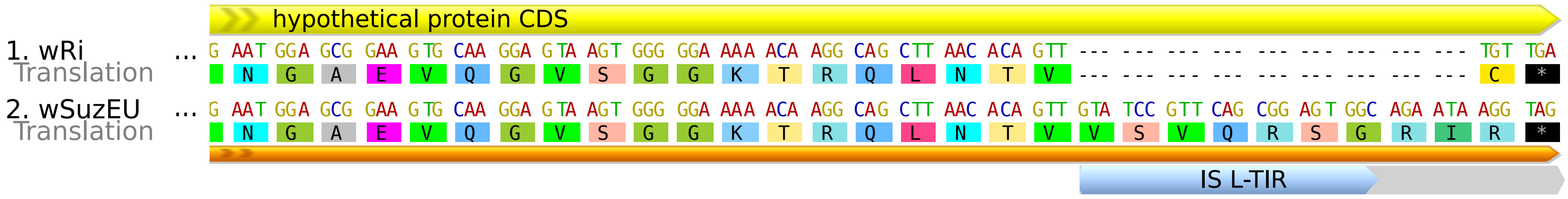

Figure S2. Full-length gel pictures.

Following gels correspond to IS and GR polymorphism from Figures 2A-2E and 3A, 3B in the main text. Lanes 1–10: Marker (bp) GeneRuler DNA Ladder Mix (Fermentas Life Sciences), *w*Ri, *w*Spc, *w*Suz_ITA, *w*Suz_FRA, *w*Suz_JPN, *w*Suz_CHN, *w*Suz_USA, *w*Suz_CAN and STC-*Wolbachia* negative control.

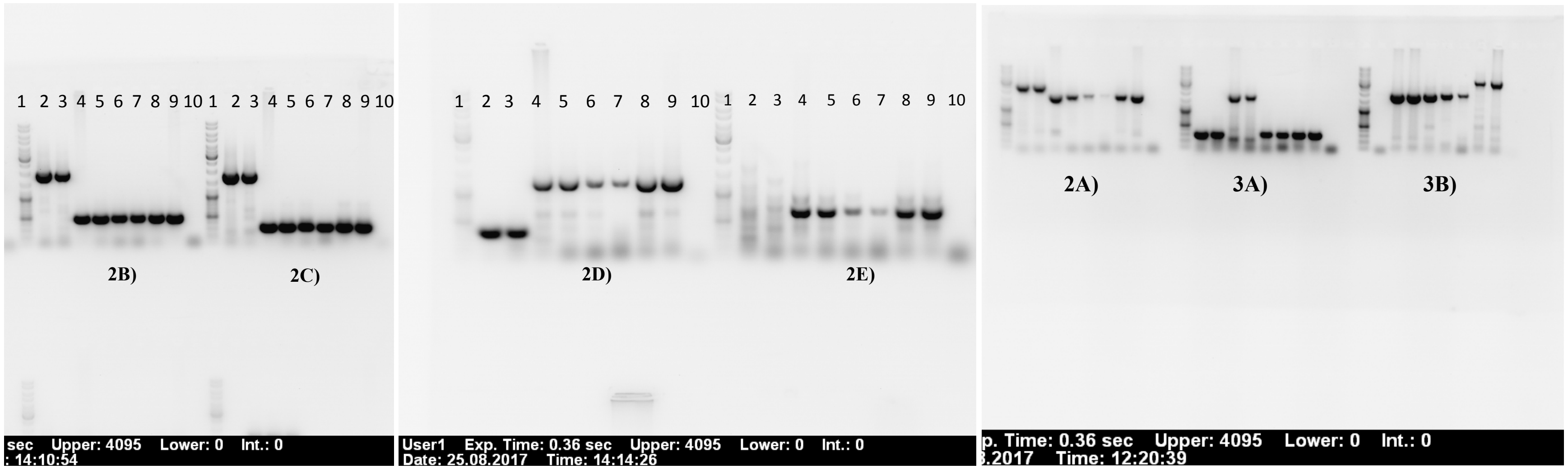

1. Marker
2. wRi
3. wSpc
4. wSuz_ITA
5. wSuz_FRA
6. wSuz_JPN
7. wSuz_CHN
8. wSuz_USA
9. wSuz_CAN
10. STC
